# The tRNA recognition mechanism of the minimalist SPOUT methyltransferase, TrmL

**DOI:** 10.1093/nar/gkt568

**Published:** 2013-06-25

**Authors:** Ru-Juan Liu, Mi Zhou, Zhi-Peng Fang, Meng Wang, Xiao-Long Zhou, En-Duo Wang

**Affiliations:** Center for RNA research, State Key Laboratory of Molecular Biology, Institute of Biochemistry and Cell Biology, Shanghai Institutes for Biological Sciences, The Chinese Academy of Sciences, 320 Yue Yang Road, Shanghai 200031, China

## Abstract

Unlike other transfer RNAs (tRNA)-modifying enzymes from the SPOUT methyltransferase superfamily, the tRNA (Um34/Cm34) methyltransferase TrmL lacks the usual extension domain for tRNA binding and consists only of a SPOUT domain. Both the catalytic and tRNA recognition mechanisms of this enzyme remain elusive. By using tRNAs purified from an *Escherichia coli* strain with the TrmL gene deleted, we found that TrmL can independently catalyze the methyl transfer from S-adenosyl-L-methionine to 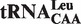 and 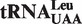 isoacceptors without the involvement of other tRNA-binding proteins. We have solved the crystal structures of TrmL in apo form and in complex with S-adenosyl-homocysteine and identified the cofactor binding site and a possible active site. Methyltransferase activity and tRNA-binding affinity of TrmL mutants were measured to identify residues important for tRNA binding of TrmL. Our results suggest that TrmL functions as a homodimer by using the conserved C-terminal half of the SPOUT domain for catalysis, whereas residues from the less-conserved N-terminal half of the other subunit participate in tRNA recognition.

## INTRODUCTION

Posttranscriptional modifications of transfer RNAs (tRNAs) are commonplace among the three kingdoms of life. Large amount of genes are involved in tRNA modification, between 1 and 10% of the genes in a given genome encode enzymes involved in tRNA modifications (1–6). The genes involved in tRNA modification outnumber the genes encoding the actual tRNAs, which indicates an extremely important role for these enzymes (1). The basic functions of tRNA modifications can be arranged into three classes (7). First, modifications in or around the anticodon loop improve the accuracy of decoding (8–10). Second, modifications in the main body affect the folding and stability of tRNAs (11–13). Third, various other modifications affect the tRNA identity (14–17). Besides directly affecting tRNA function, tRNA modifications have been shown to play regulatory roles such as responding to cellular stress, cancer or other diseases (18,19).

Most tRNA modifications were identified in the 1970s (1). Recently, however, nearly all the tRNA-modifying enzymes in model organisms such as *Escherichia coli* and *Saccharomyces cerevisiae* have been identified [RNA modification databases: http://rna-mdb.cas.albany.edu/RNAmods/ (20); http://modomics.genesilico.pl/ (21)]. The enzyme responsible for 2′-O-methylation at the 34 nt wobble position of the isoacceptors 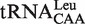 and 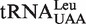 in *E. coli* was recently identified by mass spectrometry and designated TrmL (22). Deletion of TrmL in *E. coli* results in reduced efficiency of codon–wobble base interaction and affects recovery of cells from the stationary phase (22).

SPOUT represents a class of S-adenosyl-L-methionine (SAM)-dependent methyltransferases (MTases) (23,24). In 2002, Anantharaman *et al.* (23) first identified homology between the tRNA(Gm18) methyltransferase (SpoU, also named TrmH) and tRNA(m^1^G37) methyltransferase (TrmD) families and further defined the SPOUT (SpoU-TrmD) MTases superfamily. This was an important discovery owing to the low sequence similarity between SPOUT MTases, and crystal structures of SPOUT MTases were not available at that time. However, several crystal structures of SPOUT MTases including TrmH (25) and TrmDs (26–28) have since been solved and have confirmed the homology between SPOUT MTases. All available structures of SPOUT MTases contain a common catalytic domain (SPOUT domain), which exhibits an unusual alpha/beta fold with a deep topological knot in the C-terminal half (25–29). Despite their ubiquitous nature, only a few SPOUT members have been functionally characterized (24). Of these, most are involved in posttranscriptional RNA modification by methylating the ribose or base moiety of tRNA or rRNA (24). It was thought that SPOUT MTases acted specifically on RNA; however, a recent study described a novel SPOUT MTase (Yor021c) that recognizes and methylates arginine residues on proteins (30).

Although the SPOUT domain contains a highly conserved structural fold, the amino acid sequences are not conserved throughout the SPOUT superfamily, and the specificity of substrate recognition cannot be predicted based on sequence or structural homology. Although the structures of multiple SPOUT MTases have been solved, there is currently no available structure of a SPOUT enzyme in complex with substrate, until recently, the crystal structure of TrmD dimer complex with a tRNA substrate was reported at the last tRNA Conference (31); therefore, the elucidation of the mechanism of substrate recognition results primarily from biochemical studies (32–37). Most SPOUT enzymes harbor C-terminal or N-terminal extensions, which serve to bind their RNA substrates (24,33). However, there is a group of minimalist SPOUT members, which contain only the catalytic SPOUT domain and lack the extension domains. This group includes the TrmL subfamily (22), RlmH subfamily for N3 methylpseudouridine at position 1915 in 23S ribosomal RNAs (38,39) and several other uncharacterized subfamilies (24). The enzymatic characterization and substrate recognition abilities of these smallest SPOUT MTases have not yet been investigated.

**Figure 1.**
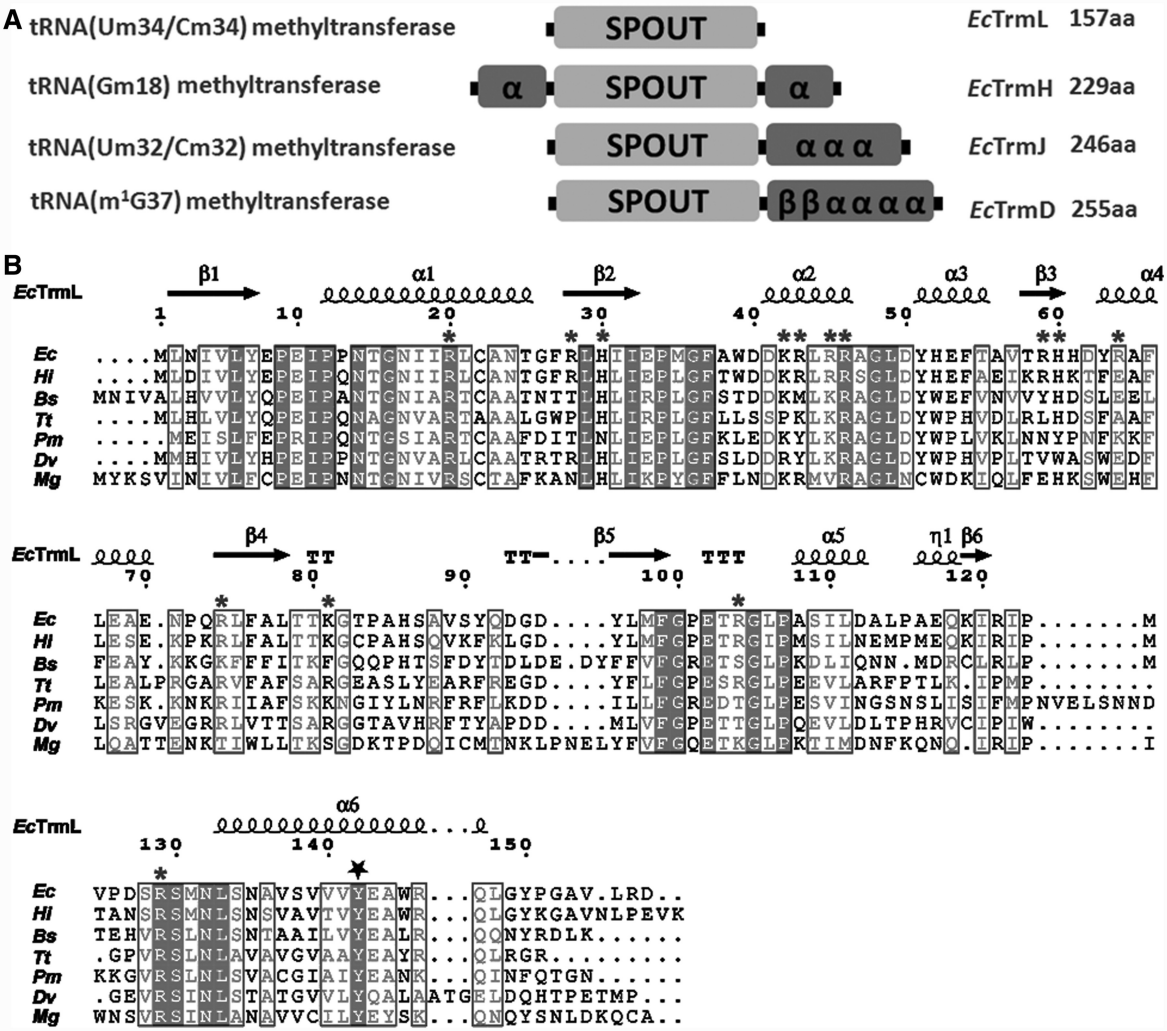
Domain architectures of SPOUT tRNA MTases and the sequence alignment of TrmLs. (**A**) The common catalytic domain of SPOUT superfamily is represented as SPOUT, the extension domains are represented by secondary structures, and the amino acid length of the respective SPOUT MTase from *E. coli* are labeled. (**B**) Structure-based multiple amino acid sequence alignment of TrmLs from model organisms. *Ec*, *E. coli*; *Hi*, *H. influenza*; *Bs*, *Bacillus subtilis*; *Tt*, *T. thermophilus*; *Pm*, *Prochlorococcus marinus*; Dv, *Desulfovibrio vulgaris*; *Mg*, *Mycoplasma genitalium*. The secondary structure elements of *Ec*TrmL are labeled above the alignment. The basic amino acid residues on the protein surface that are manipulated in this study are marked with a star. The conserved Tyr142 is marked by a pentagon.

In *E. coli*, thus far, four tRNA modification enzymes, TrmH (40,41), TrmD (42–44), TrmJ [tRNA (Um32/Cm32) methyltransferase] (45) and TrmL [tRNA (Um34/Cm34) methyltransferase] (22) have been indentified as members of the SPOUT superfamily; TrmH, TrmJ and TrmL belong to the SpoU family, and TrmD belongs to the TrmD family. All four enzymes contain a conserved SPOUT domain of ∼150 amino acid residues as shown in Figure 1A. With the exception of TrmL, they all contain extension sequences (from 70 to 100 amino acids) for tRNA binding. The domain architectures of TrmL, TrmD, TrmJ and TrmH from *E. coli* (*Ec*TrmL, *Ec*TrmD, *Ec*TrmJ and *Ec*TrmH) are shown in Figure 1A. The C-terminal extension of *Ec*TrmD consists of four α-helices and two β-strands, whereas that of *Ec*TrmJ conatins just three α-helices. In contrast, the extension domain of *Ec*TrmH is formed by two α-helices at both the N- and C-terminal ends of the SPOUT domain (Figure 1A). The structure of TrmL (also known as Yibk) from *Haemophilus influenza* confirmed that TrmL is composed only of the catalytic SPOUT domain (PDB: 1MXI and 1J85, 29). TrmL enzymes are widely distributed throughout the bacterial kingdom, and their average length is ∼150 amino acids, which suggests that the extension domains for tRNA binding are absent in all the TrmL enzymes. The sequence alignment of TrmLs from several model organisms are shown in Figure 1B.

Considering the absence of RNA binding domains in TrmL, it was hypothesized that the methylation reaction requires a protein partner to support RNA binding (29). It was later demonstrated that *Ec*TrmL failed to methylate 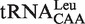 transcripts *in vitro* (45). More recently, *in vitro* methyltransferase activity of *Ec*TrmL could be detected using a long RNA chimera (∼170 nt) containing tRNA^Leu^ purified from *E. coli* as a substrate (22).

Here, we demonstrate that *Ec*TrmL alone can efficiently methylate both 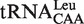 and 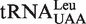 isoacceptors by using *in vivo*-purified tRNA substrates with certain modifications. By solving the crystal stuctures of *Ec*TrmL in apo form (2.0 Å) and in complex with S-adenosyl-homocysteine (AdoHcy, SAH) (2.0 Å), we identified the cofactor binding pocket and the potential active site. The catalytic activity and the tRNA recognition requirements of *Ec*TrmL were further studied by structure-based site-directed mutagensis combined with electrophoretic mobility shift assay and kinetic data. Our results demontrate that the minimalist SPOUT Mtase *Ec*TrmL functions as a homodimer by engaging residues from both subunits to bind tRNA. This work may help to elucidate the biochemical mechanism of other minimalist SPOUT MTases.

## MATERIALS AND METHODS

### Materials

[Methyl-^3^H] SAM and [^3^H] L-leucine were purchased from PerkinElmer Inc. (Waltham, MA, USA); SAM, SAH, L-leucine, dithiothreitol, NTPs, 5′-GMP, pyrophosphate, Tris-base, β-mercaptoethanol (β-Me), MgCl_2_, NaCl and KCl, and the reagents used to optimize crystallization conditions were purchased from Sigma-Aldrich Co. LLC. (St. Louis, MO, USA). Crystallization kits were from Hampton research (Aliso Viejo, CA, USA). Primers for PCR were synthesized by Invitrogen (Shanghai, China); Nickel-nitrilotriacetic (Ni-NTA) Superflow was purchased from Qiagen Inc. (Germany). KOD-plus mutagenesis kit, Pyrobest DNA polymerase and the dNTP mixture were obtained from Takara (Japan). The pET30b vector was from MerckMillipore (Darmstadt, Germany). The *E. coli* JW3581-1 strain in which the gene of TrmL was deleted (46) was purchased from the *E. coli* genetic stock center (Yale University, New Haven, CT, USA). In all, 3 mm filter papers and Superdex™ 75 column were from GE Healthcare.

### Preparation of tRNAs

Unmodified tRNAs were made by *in vitro* transcription by T7 RNA polymerase as described previously (47). Transcripts were purified by urea denaturing 12% polyacrylamide gel electrophoresis (PAGE) followed by elution. Finally, tRNAs were refolded by fast heating and slow cooling down in the presence of 5 mM MgCl_2_. The transcript of the tRNA^Leu^ was named 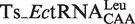 and 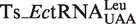, respectively.

Purification of *in vivo* expressed tRNA^Leu^ was performed as following. Plasmids expressing 
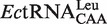
 and 
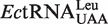
 were introduced into *E. coli* MT102 or JW3581-1, which lacks the TrmL gene. After induction with 0.5 mM Isopropyl β-D-1-thiogalactopyranoside for 12 h, cells were harvested, and crude tRNA was isolated by the standard phenol extraction procedure (48). The resulting tRNA preparations exhibited a 5–10-fold higher leucine-accepting activity than the crude tRNAs isolated from control *E. coli* strain without tRNA^Leu^ overexpression. The tRNA preparations were further purified by DEAE-Sepharose chromatography, urea denaturing PAGE and C18 reversed-phase high pressure (or high performance) liquid chromatography chromatography. At each step, denaturing PAGE, native PAGE and a leucine-accepting activity assay were used to monitor the purification of tRNA^Leu^. The tRNA^Leu^ obtained from JW3581-1 was named 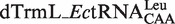 and 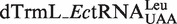, respectively.

### Purification of *Ec*TrmL and its mutants

The gene encoding *Ec*TrmL was amplified from the genome of *E. coli* MT102. Site-directed mutagenesis of *Ec*TrmL was performed by the KOD-plus mutagenesis kit as previously described (48). The genes encoding wild-type and mutant *Ec*TrmLs were cloned into vector pET30b and expressed in *E. coli* BL21 (DE3). The proteins were purified by affinity chromatography on a Ni-NTA Superflow resin, followed by gel filtration chromatography with a Superdex™ 75 column.

### Methyltransferase activity assay

The methyltransferase activity of wild-type and mutant *Ec*TrmLs for 
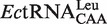
 and 
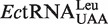
 were measured in reactions containing 100 mM Tris–HCl (pH 8.0), 150 mM KCl, 10 mM MgCl_2_, 1 mM dithiothreitol, 7 µM *Ec*tRNA^Leu^ and 20 µM [^3^H] SAM at 37°C. Reactions were initiated by adding wild-type or mutant *Ec*TrmLs (0.5 µM). For measuring the total level of methylation, 1 µM enzymes were applied. At various time intervals, aliquots were quenched by spotting on filters and washed with 5% trichloroacetic acid. The amount of radioactive [^3^H]-methyl-tRNA was measured in a Beckman Ls6500 scintillation counting apparatus. The kinetic parameters for the methylation reactions of 
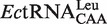
 and 
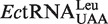
 were determined by using a range of 0.2–50 µM tRNA and 0.2 µM *Ec*TrmL. For mutants that exhibit extremely low activities, higher concentration of enzyme and tRNA substrates were used.

### Protein crystallization, structure determination and refinement

Purified *Ec*TrmL was concentrated to 12 mg/ml for crystallization. The initial crystallization conditions were screened using Index from Hampton Research. After optimizing the conditions, good crystals of *Ec*TrmL were obtained by the hanging drop vapor diffusion method at 20°C after a week under conditions of 19–22% polyethylene glycol 3350, 0.1 M 4-(2-Hydroxyethyl)-1-piperazineethanesulfonic acid [HEPES (pH 7.0)]. *Ec*TrmL and SAH were co-crystallized under conditions of 25% polyethylene glycol 4000, 5% glycerol and 0.1 M HEPES at pH 7.5 after 7 days at 16°C.

The crystals were mounted on a nylon loop and flash-cooled into a liquid N_2_ stream (−170°C) by using paraffin oil as a cryoprotectant. All crystal diffraction data sets were collected at the Shanghai Synchrotron Radiation Facility beamline BL-17U1 under 100 K. The diffraction data were processed using the HKL2000 program package (49).

The structure of *Ec*TrmL was solved by the molecular replacement method implemented in the program MOLREP (50) of the CCP4 suite (51) using the structure of the *H.influenzae* Yibk (*Hi*Yibk, PDB entry: 1J85; 29) as the starting search model. Structure refinement was performed using the program REFMAC5 (52) and PHENIX (53). Model building was performed manually with the program COOT (54). The structure determination of *Ec*TrmL-SAH complex was carried out in a similar fashion to apo *Ec*TrmL.

Throughout the refinement, 5% of randomly chosen reflections were set aside for free *R* factor monitor. The quality of final models were evaluated by PROCHECK (55) from the CCP4 suite. All molecular graphics were generated with PyMOL (DeLano Scientific; http://www.pymol.org).

### Gel mobility shift assay

Gel mobility shift assays were carried out as previously described (32) with several modifications. In all, 80 nM 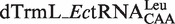 and a range of 0.75–10 μM *Ec*TrmL was incubated in a 30 μl of system of buffer A [50 mM Tris–HCl (pH 7.5), 100 mM NaCl, 5 mM MgCl_2_ and 5 mM β-Me] at 37°C for 20 min. After incubation, 3 μl of loading solution (0.25% bromophenol blue and 30% glycerol) were added into each sample and loaded immediately to a 6% polyacrylamide native gel. The electrophoresis was carried out at 4°C at a constant voltage of 60 V for 80 min, using 50 mM Tris-glycine buffer. The gel was stained with ethidium bromide for detection of RNA and using Coomassie brilliant blue to stain protein. The RNA bands were quantified by using a FujiFilm imaging analyzer.

### Isothermal titration calorimetry measurements

Isothermal titration calorimetry (ITC) measurements were performed at 25°C, using an ITC200 Micro-calorimeter (MicroCal Inc.). Experiments included 20 injections of 2 μl of SAH (1 or 1.5 mM) into the sample cell containing 100 μM proteins. The SAH and protein samples were all kept in the same buffer system which contains 20 mM Tris–HCl (pH 7.5), 100 mM NaCl, 5 mM MgCl_2_ and 5 mM β-Me. SAH titrated in an identical buffer was used as a control. Binding isotherms were fit by non-linear regression using Origin Software version 7.0 (MicroCal Inc.). The ITC data were fit to a one-site binding model using software provided by MicroCal (56,57).

### Analytical gel filtration

Analytical gel filtration was performed using a Superdex™ 75 column (10/30; column volume, 23.6 ml) in AKTApurifier Chromatograph FPLC system (GE). The buffer used for gel filtration is 20 mM Tris–HCl (pH 7.5), 100 mM NaCl, 5 mM MgCl_2_ and 5 mM β-Me. In all, 300 μl of protein samples were injected at a flow rate of 0.5 ml/min. Standard protein samples were purchased from Sigma and analyzed under the same conditions as *Ec*TrmL. The elution profiles were monitored by the absorption of UV at 280 nm.

## RESULTS

### TrmL alone catalyzes the methyl transfer from SAM to 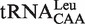 and 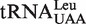 *in vitro*

**Figure 2.**
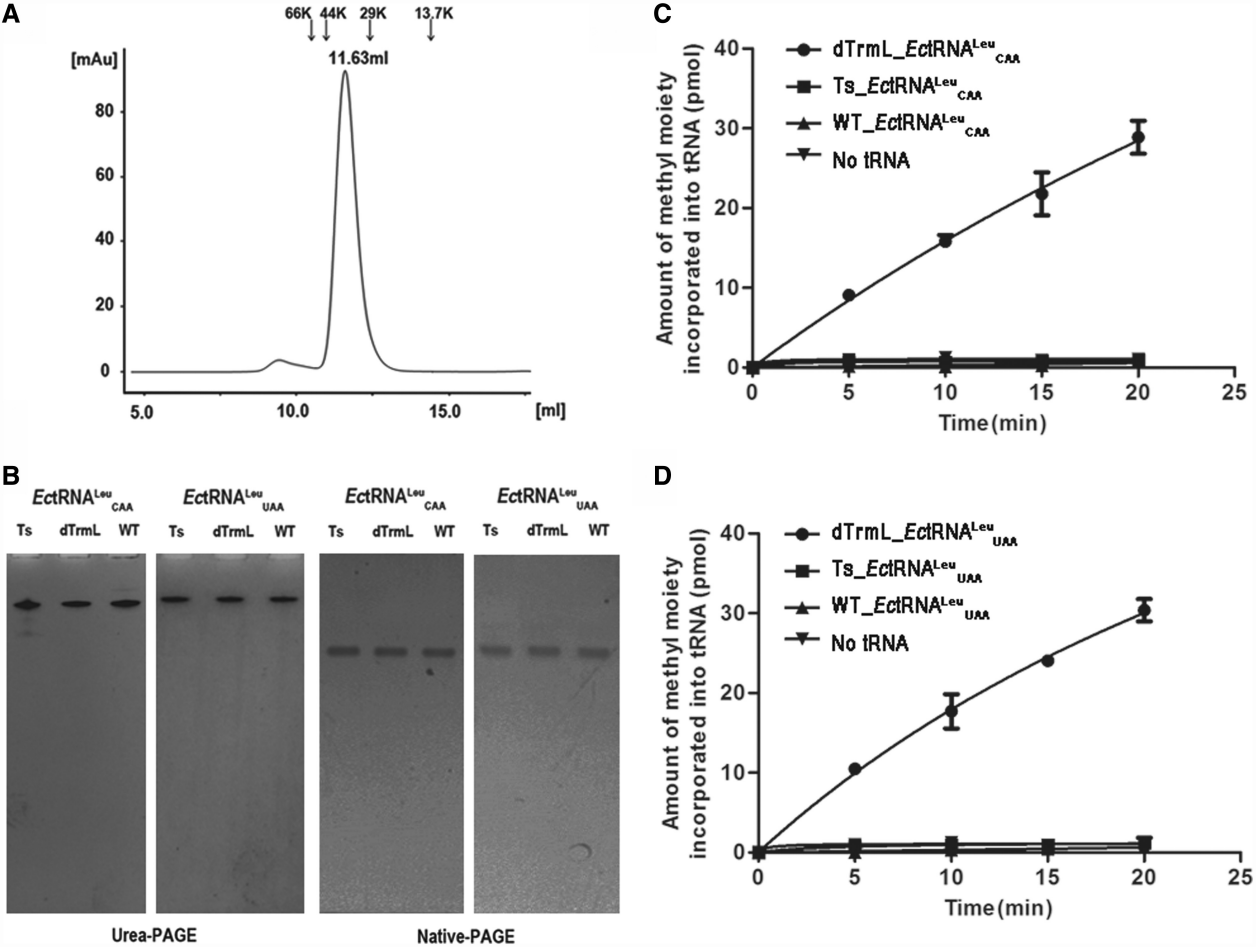
The methyltransferase activity of *Ec*TrmL. (**A**) The analytical gel filtration analyzed by superdexTM-75, *Ec*TrmL was eluted at 11.63 ml, the locations of the marker proteins are shown above the graph. (**B**) Purified tRNAs were analyzed by 12% denatured and 6% native PAGE. Ts refers to tRNA transcripts, WT and dTrmL refer to tRNAs purified from *E.coli* MT102 and JW3581-1, respectively. (**C**) and (**D**) graphically show the methyltransferase activity of *Ec*TrmL for 
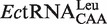
s and 
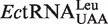
s, respectively.

To determine whether TrmL could independently catalyze the methylation of tRNA^Leu^, we performed an *in vitro* methyltransferase assay*. Ec*TrmL was purified as a dimer by analytical gel filtration chromatography. *Ec*TrmL was eluted at 11.63 ml, corresponding to a calculated molecular mass of 37.8 KDa (Figure 2A), as compared with the theoretical molecular mass of *Ec*TrmL (157 amino acids) plus linked his-tag (9 amino acids) of 18.8 KDa, suggesting that *Ec*TrmL is a dimer in solution. All fractions from this dimer peak were collected together and concentrated for the following methylation assays. For the tRNA substrates, we prepared different forms of the two isoacceptors 
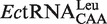
 and 
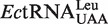
. Totally unmodified tRNAs, 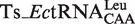 and 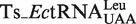, were produced by *in vitro* T7 transcription. Partially modified tRNAs, 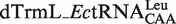 and 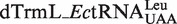, containing all other natural modifications but the modification at the target position were purified after overproduction in *E. coli* JW3581-1 deleted TrmL gene. As a control, fully modified tRNAs were purified from *E. coli* MT102. The tRNA^Leu^s were purified to homogeneity as shown by denaturing and native gel analysis (Figure 2B). These tRNAs all exhibited a leucine-accepting activity of ∼1600 pmol/A_260_ with *E. coli* leucyl-tRNA synthetase.

The tRNA transcripts and the *in vivo* purified tRNAs were then used as substrates for the methyltransferase assay. As shown in Figure 2C, *Ec*TrmL alone was able to transfer the methyl group from SAM to 
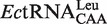
 when using the partially modified 
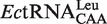
 substrate. However, there was no detectable methyltransferase activity when using the unmodified 
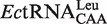
 substrate (Figure 2C). Similar results were observed with the 
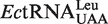
 substrate (Figure 2D). As a control, we also performed the assay on the fully modified wild-type 
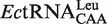
 and 
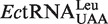
. As expected, no additional methyl groups can be transferred by *Ec*TrmL to these tRNAs (Figure 2C and D). Table 1 shows the steady-state kinetic parameters of *Ec*TrmL in the presence of various tRNA substrates. The *K*_m_ value of *Ec*TrmL for 
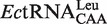
 (3.39 µM) was ∼2-fold higher than for 
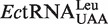
 (1.17 µM); the *k*_cat_ values of *Ec*TrmL for 
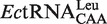
 (0.44 min^−^^1^) and 
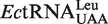
 (0.59 min^−^^1^) were similar. The reported steady state *k*_cat_ values of bacterial SPOUT tRNA MTases range from 1.0 to 15 min^−^^1^(35,36,58). The *k*_cat_ value of *Ec*TrmL(0.44 min^−^^1^ and 0.59 min^−^^1^) is much lower than that of *Streptococcus pneumoniae* TrmD [15 min^−^^1^, (36)], but in a comparable level with *Ec*TrmD [1.0 min^−^^1^, (35)] and *Tt*TrmH [1.15 min^−^^1^, (58)]. The total tRNA methylation levels by *Ec*TrmL were calculated by measuring the methylation plateau with known tRNA concentration. Both 
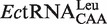
 and 
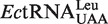
 were methylated to 80–90% in 60 min (Supplementary Figure S1), which suggest that the tRNA substrates are homogeous.

**Table 1.** Kinetic parameters of *Ec*TrmL for various tRNA^Leu^s for the methyl transfer reaction

tRNAs	*K*_m_ (μM)	*k*_cat_ (min^−1^)	*k*_cat_/*K*_m_
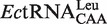 or 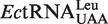 transcripts	N/A	N/A	N/A
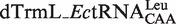	3.39 ± 0.33	0.44 ± 0.07	0.13
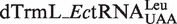	1.17 ± 0.07	0.59 ± 0.07	0.50

N/A: non-available, the results are the average of three independent repeats with standard deviations indicated.

Therefore, *Ec*TrmL alone can catalyze methyl transfer from SAM to partially modified 
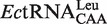
 and 
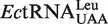
, but not to unmodified tRNA^Leu^.

### *Ec*TrmL forms a homodimer

**Figure 3.**
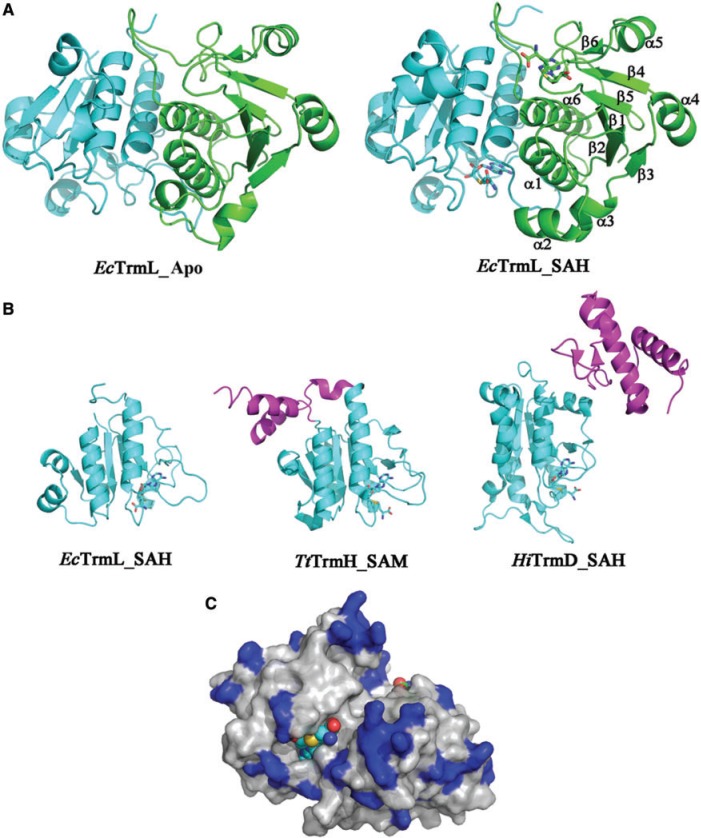
Overall structure of *Ec*TrmL. (**A**) Ribbon diagram showing the overall structure of *Ec*TrmL in Apo form (left) and in complex with SAH (right). The structures are shown as dimers, with one subunit in green and the other one in cyan. (**B**) A subunit from *Ec*TrmL, *Tt*TrmH and *Hi*TrmD are superimposed and represented from the same perspective. The common SPOUT domains are in cyan, the extensions are in magenta, SAH and SAM are shown as sticks. (**C**) Crystal structure of *Ec*TrmL with the surface colored in light gray, the basic Arg, His and Lys residues are in blue and the SAH are shown as spheres.

To understand the tRNA recognition mechanism of TrmL, we attempted to crystallize the *Ec*TrmL complexed with tRNA; however, we only obtained crystals of *Ec*TrmL alone or with a bound cofactor. We determined the tertiary structure of *Ec*TrmL in the presence and absence of SAH, which is the product of methyl transfer reaction. The final models of apo *Ec*TrmL (PDB ID: 4JAK) and the *Ec*TrmL-SAH binary complex (PDB ID: 4JAL) were both refined to 2.0 Å. Data collection parameters and refinement statistics are summarized in Table 2. The space groups of apo *Ec*TrmL and *Ec*TrmL-SAH were P3_2_ and P2_1_, respectively. Both crystals contain two molecules in one asymmetric unit, forming a tightly packed dimer (Figure 3A). Dimer formation is consistent with the observed molecular mass during analytical gel filtration. Structurally, the dimer is formed in a ‘perpendicular’ mode, i.e. one subunit (green) is rotated by ∼90 degrees with respect to the mirror molecule of the other subunit (cyan) (Figure 3A).

**Table 2.** Data collection and refinement statistics

Measurement	*Ec*TrmL	*Ec*TrmL_SAH
Data collection		
Wavelength (Å)	0.9793	0.9794
Resolution range (Å)	50.0–2.0 (2.07–2.00)	50.0–2.0 (2.07–2.00)
No. of total reflections	617 657	312 866
No. of unique reflections	42 546	19 381
I/σ	19.7 (2.8)	18.4 (2.1)
Completeness (%)	98.8 (100)	98.4 (86.5)
R_merge_	0.08 (0.53)	0.09 (0.58)
Redundancy	4.5 (4.4)	5.5 (5.0)
Space group	P3_2_	P2_1_
Unit cell dimensions		
a, b, c (Å)	84.95, 84.95,78.89	44.28, 66.29, 49.77
α, β, γ (deg)	90.00, 90.00,120.00	90.00, 90.24, 90.00
Refinement		
Resolution (Å)	50.0–2.0	49.1–2.0
R_work/_R_free_	0.189/0.204	0.212/0.245
No. of reflections	40388	18237
No. of atoms		
Protein atoms	2450	2462
Water/other	214	49/2 glycerol
SAH	0	2
HEPES	0	1
R.m.s. deviations		
Bond lengths (Å)	0.008	0.009
Bond angles (deg)	1.143	1.24
Average B factor (Å^2^)	41.2	39.6
Ramachandran statistics (%)		
Most favored	99.3	98
Allowed	0.7	2

Values in parentheses are for highest-resolution shell.

The overall structure of an *Ec*TrmL monomer subunit is composed of six β-strands and six α-helices, in the order β1-α1-β2-α2-α3-β3-α4-β4-β5-α5-β6-α6 (Figure 3A). The parallel six-stranded β-sheet is flanked by four α-helices on one side and by two α-helices on the other side (Figure 3A). The N-terminal half forms a Rossmann-like fold, which is common among SAM-dependent MTases, whereas the C-terminal half forms a deep trefoil knot (Figure 3A). The knot is formed by threading the C-terminus (residues 121–157) through a hoop composed of residues 78–86 (Figure 3A), which forms a hydrophobic core that stabilizes the polypeptide knot. The cofactor product SAH is bound at this knot region (Figure 3A). The overall structure of *Ec*TrmL is similar to the crystal structure of *Hi*Yibk (PDB: 1MXI and 1J85, 29), only with variations in loop regions.

The folding of the catalytic domain (cyan) of *Ec*TrmL is similar to other known SPOUT MTases involved in tRNA modification, including *Thermus thermophilus* TrmH (*Tt*TrmH, PDB: 1V2X) and *H. influenza* TrmD (*Hi*TrmD, PDB: 1UAL) (Figure 3B). The structure of *Ec*TrmL can be superimposed onto the catalytic domain of *Tt*TrmH with a core rmsd of 2.02 Å (136 Cαs, with 23.5% sequence identity) and to *Hi*TrmD with a core rmsd of 2.54 Å (99 Cαs), despite having a sequence identity of only 9.1%. TrmL, however, lacks the extension domains involved in tRNA binding found in *Tt*TrmH and *Hi*TrmD (Figure 3B, magenta).

*Ec*TrmL contains many basic amino acid residues, comprising 13 arginines (8.3%), three lysines (1.9%) and five histidines (3.2%) in a total 157 resides, most of which are located on the protein surface (Figure 3C). A region of positively charged basic residues is located around the SAH binding pocket as well as other distal regions.

### Cofactor binding pocket and active site of *Ec*TrmL

**Figure 4.**
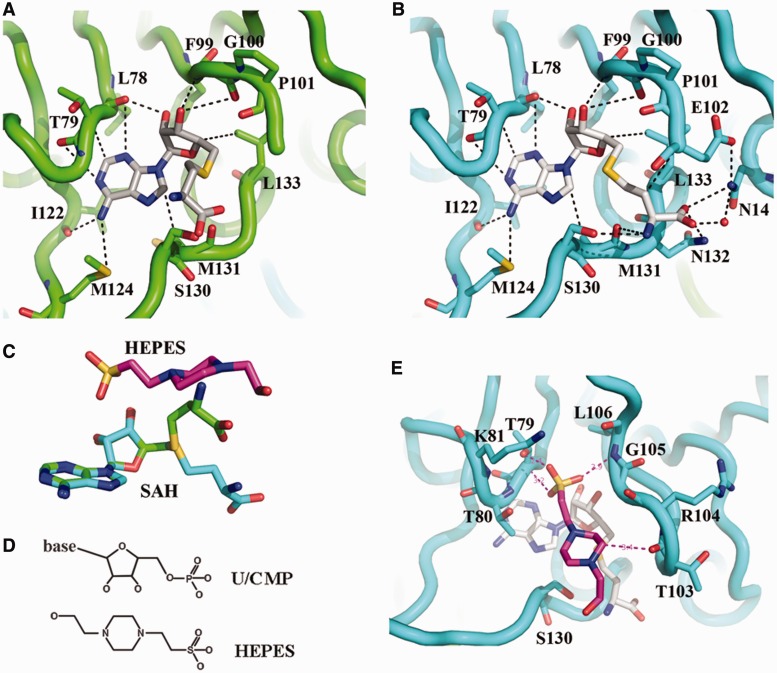
SAH and HEPES binding. (**A**) SAH bound in subunit A. The carbon atom of SAH is shown in white, the backbone of *Ec*TrmL is in green, and all the residues within 4 Å from SAH are shown in stick. (**B**) SAH binding details in subunit B, the backbone of *Ec*TrmL is shown in cyan. (**C**) The crystal structures of SAH molecules from subunit A (green) and subunit B (cyan) are superimposed and shown as sticks, with the structure of HEPES in magenta. (**D**) The chemical structure of the ribose and phosphate of U/CMP, and the HEPES molecule. (**E**) The structure of a HEPES molecule bound to *Ec*TrmL, with all the residues within 4 Å shown as sticks. The carbon atoms of HEPES and SAH are shown in magenta and white, respectively.

SAH binding does not significantly affect the overall structure of *Ec*TrmL (Figure 3A). Although both subunits have a bound SAH, the conformations of the two SAHs are strikingly different with respect to the orientation of the homocysteine groups (Figures 4A–C and Supplementary Figure S2). The adenine and ribose groups of SAH form a broad interaction network with the cofactor binding pocket formed primarily by residues from three loops (residues 78–80; 99–106; 120–124) (Figure 4A and B), and their conformations are exactly the same in both subunits. The SAH in subunit A is in the characteristic bent conformation (Figure 4A) already observed for SAH and SAM found in *Tt*TrmH (25), *Hi*Yibk (29) and TrmDs (26,28). The homocysteine group of SAH points toward the entrance of the catalytic crevice, which does not have many interactions with *Ec*TrmL (Figure 4A). Although in subunit B, the homocysteine group of SAH is located near to residues Asn14, Glu102 and 130–133 (Figure 4B). The amine group of homocysteine forms hydrogen bonds with the side chain of Ser130 and the carboxyl group of Met131. The carboxyl group of homocysteine forms hydrogen bonds with residues Asn14, Asn132 and Leu133. The Asn14 is conserved throughout the SpoU family and was reported to be involved in the release of SAH (58).

In subunit B, a HEPES molecule from the crystal growth buffer is observed on top of SAH in the potential active site and is well stabilized with high density occupancy (Supplementary Figure S2). From the chemical structure (Figure 4D), the HEPES molecule is similar to the ribose and the phosphate group, which suggest that HEPES may mimic the U34 or C34 of the tRNA substrate in this context. The HEPES molecule is involved in a complex interaction network with residues 79–81, 103–106, Ser130 and SAH (Figure 4E). The residues involved in HEPES binding are consistent with the putative active site for the SpoU family MTases (25). The HEPES molecule in subunit B is located in the same position as the homocysteine group of SAH in subunit A (Figure 4C). This suggests that in subunit B, the structure is in a state in which the SAH and the tRNA substrate are both bound. The wider interaction network for SAH binding in subunit B provides structural evidence that these residues comprise the active site.

From the crystal structures of *Ec*TrmL with SAH and HEPES, the SAH binding pocket and the active site are primarily formed by residues from the C-terminal half of the subunit. Three loops (residues 78–80, 99–106 and 120–124) form the SAH binding pocket, and on tRNA substrate binding, loops comprising residues 79–81, 103–106 and 130–133 form the active site. Asn14 from the N-terminal half is also involved in binding with SAH in this state.

### Glu scanning of basic amino acid residues on the protein surface

Our results show that *Ec*TrmL can independently catalyze the methylation of tRNA^Leu^, despite lacking the usual extension domain for tRNA binding. However, the tRNA binding mechanism of *Ec*TrmL remains unclear. From the tertiary structure, a high basic amino acid content was observed on the surface of *Ec*TrmL. Considering the negative charge of phosphate groups in the tRNA substrate, we screened for residues involved in tRNA binding by mutating the basic amino acid residues on the surface of *Ec*TrmL to the negatively charged amino acid Glu. When selecting residues for mutagenesis, we tried to avoid residues that may be involved in SAH binding or in dimer formation. Finally, 14 residues (Arg20, 28, 43, 45, 46, 59, 64, 74, 104, 129; Lys42 and 81; His30 and 60) were selected. With the exception of *Ec*TrmL-H30E and -H60E, all of the single mutants were soluble and exhibited a dimer structure in solution.

**Figure 5.**
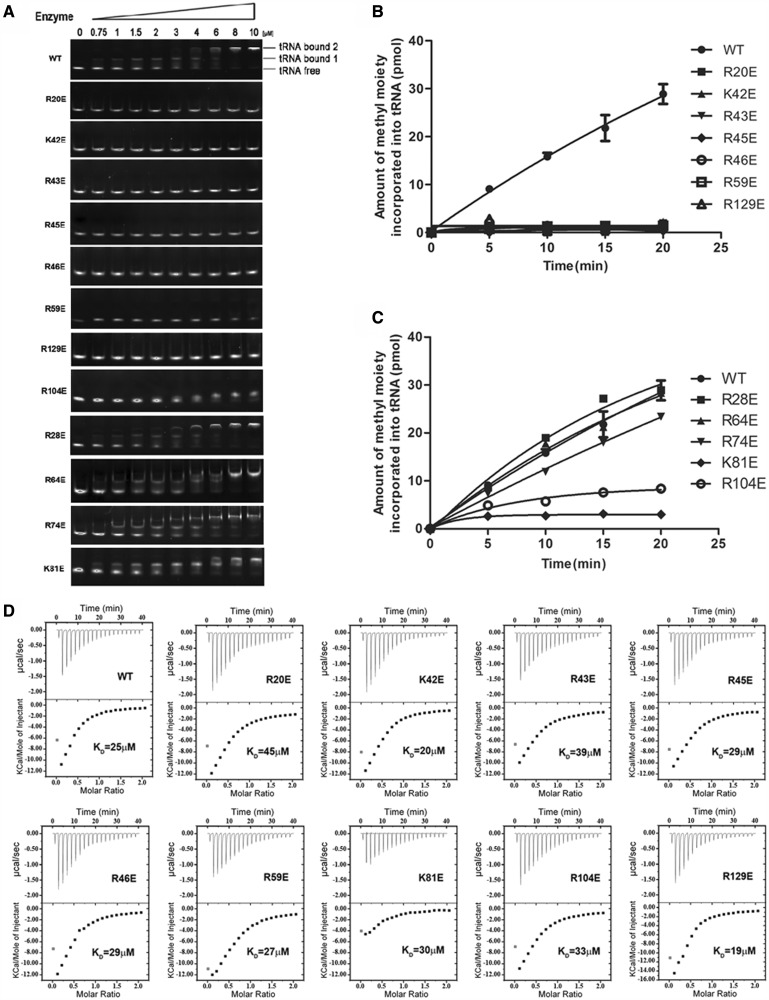
Glu scanning of the basic amino acid surface residues. (**A**) The binding affinities of *Ec*TrmLs for tRNA analyzed by the gel mobility shift assay. (**B**) and (**C**) show the methyltransferase activities of the various *Ec*TrmL mutants. (**D**) The SAH-binding affinity as measured by ITC.

We first analyzed the affinities of the wild-type and mutant *Ec*TrmLs for tRNA by the gel mobility shift assay (Figure 5A). In all, 80 nM 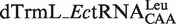 or ^32^P-labeled 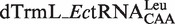 was used in the system (Figure 5A and Supplementary Figure S3). For wild-type *Ec*TrmL, a shift was observed starting at 0.75 μM enzyme representing the *Ec*TrmL-tRNA complex (tRNA bound 1). A larger molecular mass complex or aggregate was represented by a supershift (tRNA bound 2) above 4 μM *Ec*TrmL (Figure 5A and Supplementary Figure S3). A similar high-molecular mass complex was reported in a previous study performed on *Tt*TrmH (58). The *K_d_* value was calculated by quantifying the intensities of the lower *Ec*TrmL-tRNA complex band. Owing to the formation of the aggregate complex at high protein concentration, it was difficult to precisely determine the *K*_d_ value, although it was estimated to be ∼1.9 μM. The tRNA binding affinities of *Ec*TrmLs are compared in Figure 5A. The mutants can be divided into three groups. Mutants in the first group (*Ec*TrmL-R20E, -K42E, -R43E, -R45E, -R46E, -R59E and -R129E) showed no detectable binding to tRNA (Figure 5A). Mutants in the second group (*Ec*TrmL-R104E) exhibited decreased binding affinity to tRNA as compared with *Ec*TrmL, and mutants in the last group (*Ec*TrmL-R28E, -R64E, -R74E and -K81E) had a similar binding affinity for tRNA as wild-type *Ec*TrmL (Figure 5A).

The methyltransferase activity of the wild-type and mutant *Ec*TrmLs was measured, and the results are shown in Figure 5B and C. The majority of mutants display a methyltransferase activity that is consistent with their tRNA binding affinity. All of the mutants in the first group that did not bind tRNA showed no detectable methyltransferase activity (Figure 5B). Similarly, *Ec*TrmL-R104E of the second group showed a reduction of tRNA-binding affinity, with an associated decreased in methyltransferase activity (Figure 5C). In the last group, *Ec*TrmL-R28E, -R64E and -R74E had the same methyltransferase activity as *Ec*TrmL, which was consistent with their tRNA-binding affinity (Figure 5C). The exception was *Ec*TrmL-K81E, which efficiently bound tRNA showed greatly reduced catalytic activity. This mutant was selected for further study.

We then used ITC to measure the SAH-binding affinity of all the mutants with reduced catalytic activity (Figure 5D). The results showed that *Ec*TrmL-R20E, -K42E, -R43E, -R45E, -R46E, -R59E, -R104E and -R129E had dissociation constants (K_D_) of SAH comparable to *Ec*TrmL, suggesting that the defects in methyltransferase activity were not caused by reduced SAH binding, but instead from reduced tRNA binding. Again, *Ec*TrmL-K81E represents an exception, as the binding affinity of SAH was similar to *Ec*TrmL (Figure 5D), but the enthalpy change on SAH binding was noticeably decreased compared with that of *Ec*TrmL or the other mutants. As the methyltransferase activity of *Ec*TrmL-K81E was too low to solve the kinetic parameters, we made an Ala mutation to analyze the function of K81. The kinetic parameters of *Ec*TrmL-K81A are shown in Supplementary Table S1. Compared with *Ec*TrmL, the *K_m_* value for tRNA remains unchanged, but the *k*_cat_ value is 40% lower than *Ec*TrmL, suggesting that K81 is involved in catalysis, but not in tRNA binding.

### Ala scanning identifies residues required for tRNA recognition

**Figure 6.**
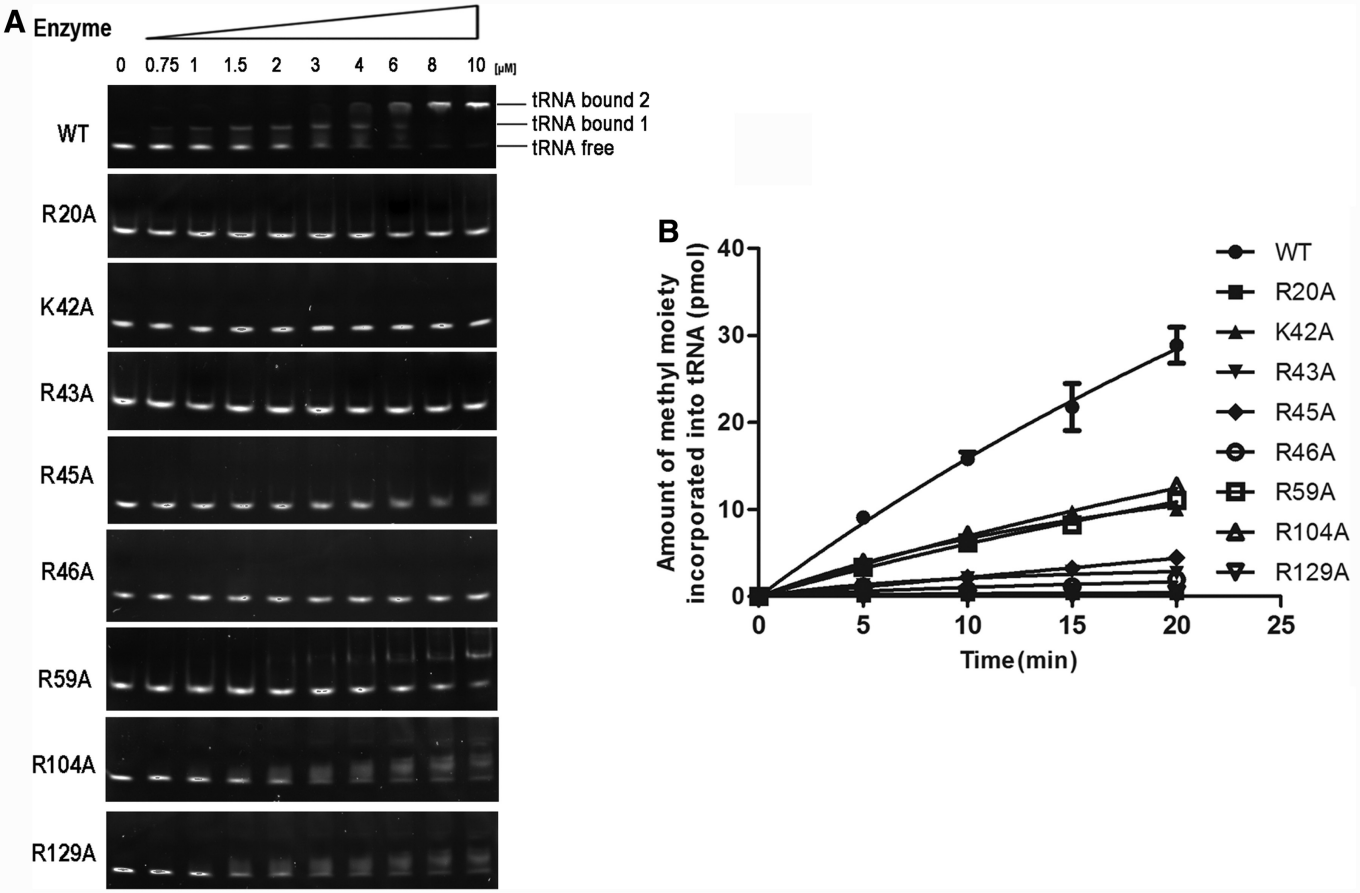
Ala mutation of residues involved in tRNA binding. (**A**) The binding affinities of *Ec*TrmLs for 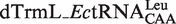 analyzed by the gel mobility shift assay. (**B**) The methyltransferase activities of the various *Ec*TrmL mutants.

To further understand the role of the basic residues involved in tRNA binding, we made Ala mutations of the residues described earlier in the text and further analyzed their properties. All of the mutants showed decreased (*Ec*TrmL-R45A, -R59A, -R104A and -R129A) or not-detectable affinity to tRNA (*Ec*TrmL-R20A, -K42A,-R43A and -R46A), as measured by the gel mobility shift assay (Figure 6A). All of the mutants showed reduced methyltransferase activity compared with *Ec*TrmL, whereas *Ec*TrmL-R20A, -R43A, -R46A and -R129A had no detectable catalytic activity (Figure 6B). The *K_m_* values of *Ec*TrmL-R104A, -R59A, -K42A and -R45A, for tRNA were 3–20-fold higher than that of *Ec*TrmL while their *k*_cat_ values did not change significantly (Table 3). These results suggest that the loss of catalytic activity of these mutants is due to their low affinity for tRNA, and this is consistent with the results from the gel mobility shift assay.

**Table 3.** tRNA-binding affinity and kinetic parameters of wild-type and mutant *Ec*TrmL

*Ec*TrmL variants	Affinity for tRNA by electrophoretic mobility shift assay	*K*_m_ (μM)	*k*_cat_ (min^−1^)	*k*_cat_/*K*_m_ (relative)
Wild type	++	3.39 ± 0.33	0.44 ± 0.07	1
R20A	+−	N/D	N/D	N/D
K42A	+−	26.9 ± 11.7	0.62 ± 0.18	0.17
R43A	−	N/D	N/D	N/D
R45A	+−	58.5 ± 7.6	0.37 ± 0.04	0.05
R46A	−	N/D	N/D	N/D
R59A	+−	11.17 ± 1.59	0.43 ± 0.13	0.29
R104A	+	9.78 ± 1.55	0.55 ± 0.21	0.42
R129A	+	N/D	N/D	N/D

N/D: not detectable, the results are the average of three independent repeats with standard deviations indicated.

### *Ec*TrmL dimer formation is essential for tRNA recognition

**Figure 7.**
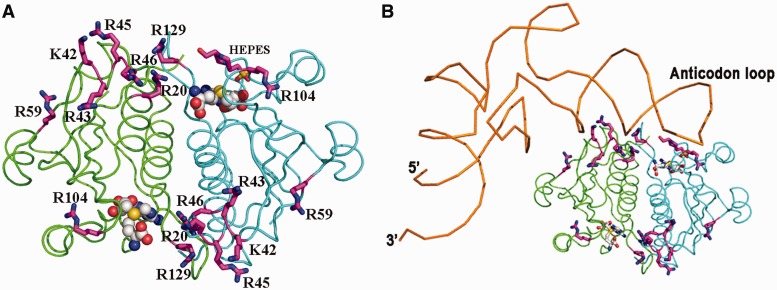
The proposed model of tRNA^Leu^ binding to *Ec*TrmL. (**A**) The *Ec*TrmL basic amino acid surface residues (magenta) that are identified as being involved in tRNA binding, the dimer structure is shown in cartoon loop with same color used as in Figure 3. (**B**) The proposed model of *Ec*TrmL bound with tRNA, the backbone of 
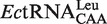
 is shown in brown.

The basic residues involved in tRNA binding are shown in Figure 7A (magenta). These residues appear to form a positively charged patch, which could represent the tRNA-binding site. The positively charged patch includes residues R104 and R129 near the active site from one subunit, and residues R20, K42, R43, R45, R46 and R59 from the other subunit (Figure 7A). It therefore appears that tRNA binding requires the cooperation of both subunits of the dimer. Based on our unpublished data, the nucleotide residues in the anticodon loop of the tRNA are important for recognition by *Ec*TrmL. The proposed model of *Ec*TrmL of cooperative tRNA binding by both dimer subunits is presented in Figure 7B. According to this model, residues R20, K42, R43, R45, R46 and R59 from the N-terminal half of the SPOUT domain appear to substitute for the extension domains present in TrmH and TrmD.

**Figure 8.**
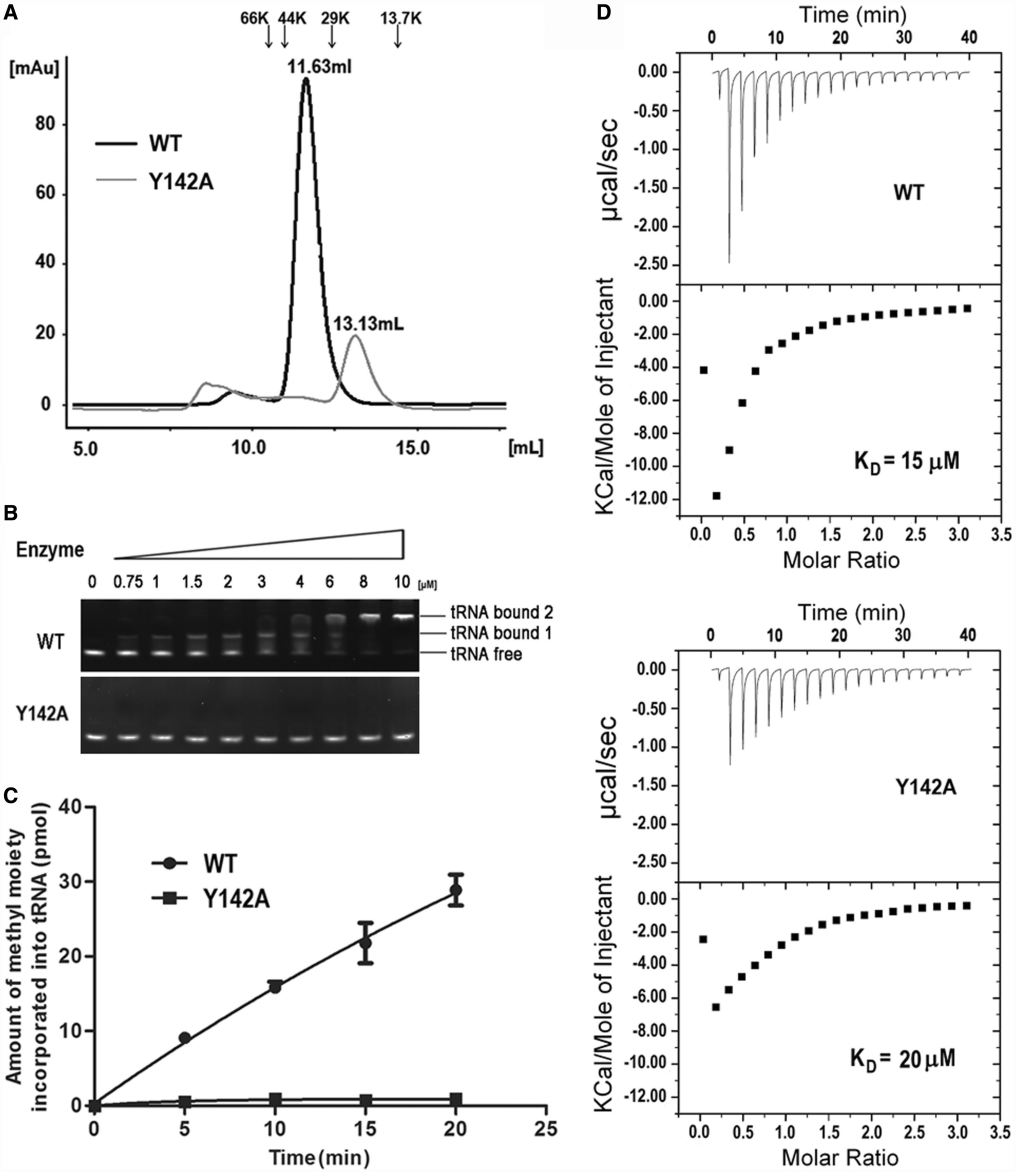
The effect of dimer formation on SAH and tRNA binding and enzymatic activity. (**A**) The analytical gel filtration of *Ec*TrmL-Y142A was analyzed by under the same conditions as wild-type *Ec*TrmL and was eluted at 13.13 ml. (**B**) The binding affinity of WT and Y142A for 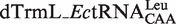 was analyzed by the gel mobility shift assay. (**C**) The methyltransferase activity of Y142A. (**D**) The binding affinity for SAH by ITC.

Based on the proposed model, the tRNA is bound by residues from both subunits. However, there still remains the possibility that the tRNA binds only one subunit, in which case the dissociation of the subunits should not affect tRNA binding. We analyzed the dimer interface (Supplementary Figure S4) and identified the most important interactions between the subunits. Ala substitutions of the residues at the dimer interface were performed to disrupt the interactions and cause dissociation of the dimer structure. Only the mutant *Ec*TrmL-Y142A could affect the oligomeric structure as shown by analytical gel filtration. The elution volume of *Ec*TrmL-Y142A through a Superdex-75 gel filtration column under the same conditions as *Ec*TrmL (Figure 8A) was 13.13 ml, which corresponds to a calculated molecular mass of 19.8 KDa, indicating that *Ec*TrmL-Y142A is a monomer in solution. The results showed that the residue Y142 is critical for maintaining the dimeric form of *Ec*TrmL, which is consistent with its central position at the interface (Supplementary Figure S4). In support of this model, Y142 is strictly conserved in the TrmL subfamily (Figure 1B).

*Ec*TrmL-Y142A failed to form a complex with tRNA in the gel mobility shift assay, suggesting that the monomeric form of *Ec*TrmL cannot form a stable complex with the tRNA (Figure 8B). In addition, the methyltransferase activity of *Ec*TrmL-Y142A was completely abrogated (Figure 8C), despite the fact that the isolated subunit could bind SAH with comparable affinity to *Ec*TrmL, as shown by ITC experiments (Figure 8D).

## DISCUSSION

The purified *Ec*TrmL alone can efficiently transfer a methyl group from SAM to both 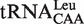 and 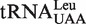 isoacceptors, but only to tRNAs, which contain certain posttranscriptional modifications, which were purified from *E. coli* strain with TrmL gene deleted. Hence, *Ec*TrmL only recognizes its tRNA substrates containing the natural modifications at other sites. These results are consistent with an earlier *in vivo* study (22), which showed that deletion of the MiaA gene, which is involved in ms^2^i^6^ modification at A37, caused loss 2′-O-methylation of U34 and C34 of 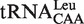 or 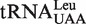, respectively (22). Therefore, the result suggests that ms^2^i^6^ modification at nucleotide A37 is one of the modifications required for *Ec*TrmL to recognize its tRNA substrates. From the proposed model of *Ec*TrmL with tRNA, residues including A37 from the tRNA anticodon loop may have potential interactions with *Ec*TrmL; the ms^2^i^6^ modification that provides extra groups on A37 base may increase the chance of having direct interaction with *Ec*TrmL. To understand the role of this modification in tRNA recognition, solving the structure of *Ec*TrmL/tRNA complex will be ideal. Recent studies showed that a lack of non-essential tRNA modifications can lead to a rapid decay of tRNA (59). As the regulation of posttranscriptional modifications of tRNA *in vivo* is extremely complex, we suggest that TrmL may serve as a checkpoint such that tRNAs lacking the necessary modifications will be excluded from the process of tRNA maturation.

The presence of a HEPES molecule in the *Ec*TrmL-SAH complex serves to mimic the U34/C34 nucleotide of the tRNA substrate and allows the opportunity to probe interactions between SAH and *Ec*TrmL and provide more information about the active site. The SAH-binding pocket and the active site are formed primarily by residues from the C-terminal half of *Ec*TrmL. This is similar to all other reported SPOUT MTases, which may explain the higher sequence conservation in the C-terminal half than in the N-terminal half of SPOUT domains (24).

We have identified a series of basic amino acid residues (R20, K42, R43, R45, R46, R59, R104 and R129) on the protein surface that are involved in the initial binding of tRNA substrates. Residues R104 and R129 are located near the active site. R129 is conserved in all TrmLs and plays a role in catalysis, as mutating R129 to Glu or Ala led to a complete loss of methyltransferase activity. Residues K42, R43, R45 and R46 are all located on helix α2 in the N-terminal half of the SPOUT domain. Structurally, the side chains of these four basic residues are exposed on the surface of *Ec*TrmL, which provides a docking region for the tRNA substrate (Figure 7A). Sequence alignment of the TrmL subfamily shows that this region (K42–R46) is mainly composed of basic amino acids (Figure 1B), and that residue R46 is strictly conserved, which suggests that helix α2 plays a crucial role in binding the tRNA substrate in all TrmLs. Residues R20 and R59 are also located in the N-terminal half of the SPOUT domain. Residue R20 is a critically conserved residue in the SpoU family. The conservation of the residues involved in tRNA binding suggests that the tRNA recognition mechanism shown here for *Ec*TrmL should be conserved throughout the TrmL subfamily.

Dimerization of *Ec*TrmL is observed both in solution and in the crystal structure, suggesting that it functions as a homodimer. The tRNA recognition motifs from the protein surface suggest that the tRNA-binding site spans the two subunits of the dimer instead of being located in a single subunit. The dissociated monomer could efficiently bind the SAH cofactor, but did not retain the tRNA-binding capacity and catalytic activity of the homodimer. These results further support the assumption that the binding of tRNA requires the cooperation of both subunits in the dimer. Therefore, *Ec*TrmL can efficiently bind tRNA by recruiting residues from the SPOUT domain of the other subunit, in the absence of the tRNA-binding extension domain. From the structure, the residues K42, R43, R45, R46 and R59 are located far from the tRNA-binding site and are all from the less conserved N-terminal half of the SPOUT domain. We propose that these basic residues play the same role as the extension domains in the other SPOUT MTases. To date, all of the studied SPOUT members have been found to be functional dimers (24). TrmH dimerizes essentially like TrmL in a ‘perpendicular’ fashion, whereas TrmD dimerizes in an ‘antiparallel’ fashion (Figure 3 and Supplementary Figure S5). When analyzing the dimeric structures of *Tt*TrmH and *Hi*TrmD (Supplementary Figure S5), it appears that the extension domains responsible for tRNA binding are oriented toward the active site of the other subunit, which suggests that the tRNA-binding sites in these enzymes should also span both subunits as in *Ec*TrmL. However, the positions of the tRNA-binding domains of *Tt*TrmH or *Hi*TrmD and the tRNA-binding motifs of TrmL are different. This may be explained by the fact that these enzymes have to recognize and accommodate different tRNA structural elements to catalyze methylation at specific positions.

The SPOUT MTases can be divided into two classes, (i) the smallest SPOUT MTases that including TrmL, RlmH and other proteins with unknown functions and consist only of the SPOUT catalytic domain and (ii) the larger SPOUT proteins that all contain N- or C-terminal appended domains involved in substrate binding. As homodimer formation occurs in all of the known SPOUT MTases, we suggest that dimer formation also occurred in the Last Universal Common Ancestor of SPOUT members. The ability of *Ec*TrmL to recruit residues from the N-terminal half of the SPOUT domain to bind the tRNA substrate may be conserved in other small SPOUT MTases and the Last Universal Common Ancestor. As each SPOUT enzyme recognizes a specific substrate, this may explain the increased sequence divergence observed in the N-terminal half of the SPOUT domain as compared with the catalytic C-terminal half. Throughout the evolution of SPOUT MTases, they were required to recognize increasingly varied substrates, including tRNAs, rRNAs and even proteins. As a result, the SPOUT MTases evolved new extension domains to expand their substrate recognition capacity as in TrmH, TrmD and other larger SPOUT members.

During catalysis, whether one or two tRNA molecules bind to the *Ec*TrmL dimer remains unclear. As the two subunits of the homodimer are identical, they should be able to simultaneously bind one tRNA in each catalytic site. In the crystal structure, only one HEPES molecule is bound, where it potentially mimics the tRNA acceptor nucleotide. Even when the amount of HEPES was increased in the crystal growth conditions, only one HEPES molecule was bound. It is therefore possible that during catalysis, only one tRNA molecule binds per *Ec*TrmL dimer. Although several crystal structures of SPOUT MTases have been reported (25–29), there is no available structure of a SPOUT MTase in complex with an RNA substrate, and the details of the interactions between these enzymes and their substrates are poorly understood. Here, we present the tRNA substrate recognition motifs of the smallest SPOUT MTase TrmL, which may contribute to the functional and structural characterization of other related SPOUT members.

## ACCESSION NUMBERS

PDB accession numbers: 4JAK, 4JAL.

## SUPPLEMENTARY DATA

Supplementary Data are available at NAR Online: Supplementary Table 1 and Supplementary Figures 1–5.

## FUNDING

Natural Science Foundation of China [31270775 and 31130064]; National Key Basic Research Foundation of China [2012CB911000]. Funding for open access charge: National Key Basic Research Foundation of China [2012CB911000].

*Conflict of interest statement*. None declared.

## Supplementary Material

Supplementary Data
