# Supplementary material for: The tRNA recognition mechanism of the minimalist SPOUT methyltransferase, TrmL
Source: Nucleic Acids Res. 2013 Jun 25;41(16):7828–42. doi: 10.1093/nar/gkt568 (PMC3763551; doi:10.1093/nar/gkt568)
Supplement: Supplementary Data [file supp_41_16_7828__index.html]

The tRNA recognition mechanism of the minimalist SPOUT methyltransferase, TrmL — The tRNA recognition mechanism of the minimalist SPOUT methyltransferase, TrmL — Supplementary Data 

# The tRNA recognition mechanism of the minimalist SPOUT methyltransferase, TrmL

## 

files

**Files in this Data Supplement:**

- Supplementary Data - doc file
