# Supplementary material for: The tRNA recognition mechanism of the minimalist SPOUT methyltransferase, TrmL
Source: Nucleic Acids Res. 2013 Jun 25;41(16):7828–42. doi: 10.1093/nar/gkt568 (PMC3763551; doi:10.1093/nar/gkt568)
Supplement: Supplementary Data [file supp_gkt568_nar-00550-r-2013-File010.doc]

**The tRNA recognition mechanism of the minimalist SPOUT methyltransferase, TrmL**

Ru-Juan Liu, Mi Zhou, Zhi-Peng Fang, Meng Wang, Xiao-Long Zhou, En-Duo Wang*

Center for RNA research, State Key Laboratory of Molecular Biology, Institute of Biochemistry and Cell Biology, Shanghai Institutes for Biological Sciences, The Chinese Academy of Sciences, 320 Yue Yang Road, Shanghai 200031, China

The authors wish it to be known that, in their opinion, the first two authors should be regarded as joint First Authors

*To whom correspondence should be addressed. Tel: +86 21 54921241; Fax: +86 21 54921011; Email: [edwang@sibcb.ac.cn](mailto:edwang@sibcb.ac.cn)

**Table S1** Kinetic parameters of wild type *Ec*TrmL and Lys81 mutants.

| *Ec*TrmL Variants | Affinity for tRNA by EMSA | *K*_m_ (μM) | *k_cat_* (min^-1^) | *k_cat_*/*K*_m_ (relative) |
| --- | --- | --- | --- | --- |
| Wild type | ++ | 3.39±0.33 | 0.44±0.07 | 1 |
| K81E | ++ | N/D | N/D | N/D |
| K81A | ++ | 2.40±0.94 | 0.23±0.07 | 0.74 |

**Figure S1**

**
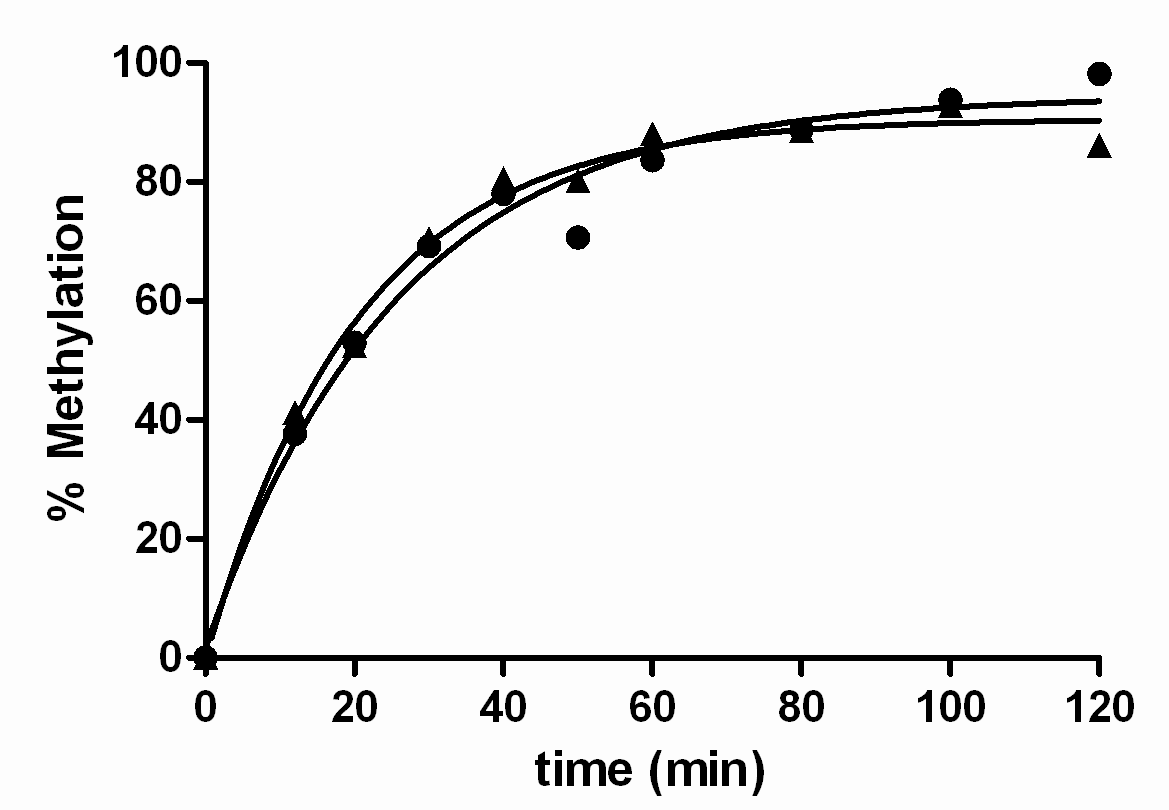
**

**Figure S1** The methyltransferase activity of *Ec*TrmL for dTrmL_*Ec*tRNA^Leu^_CAA_(●) and dTrmL_*Ec*tRNA^Leu^_UAA_(▲).

**Figure S2**


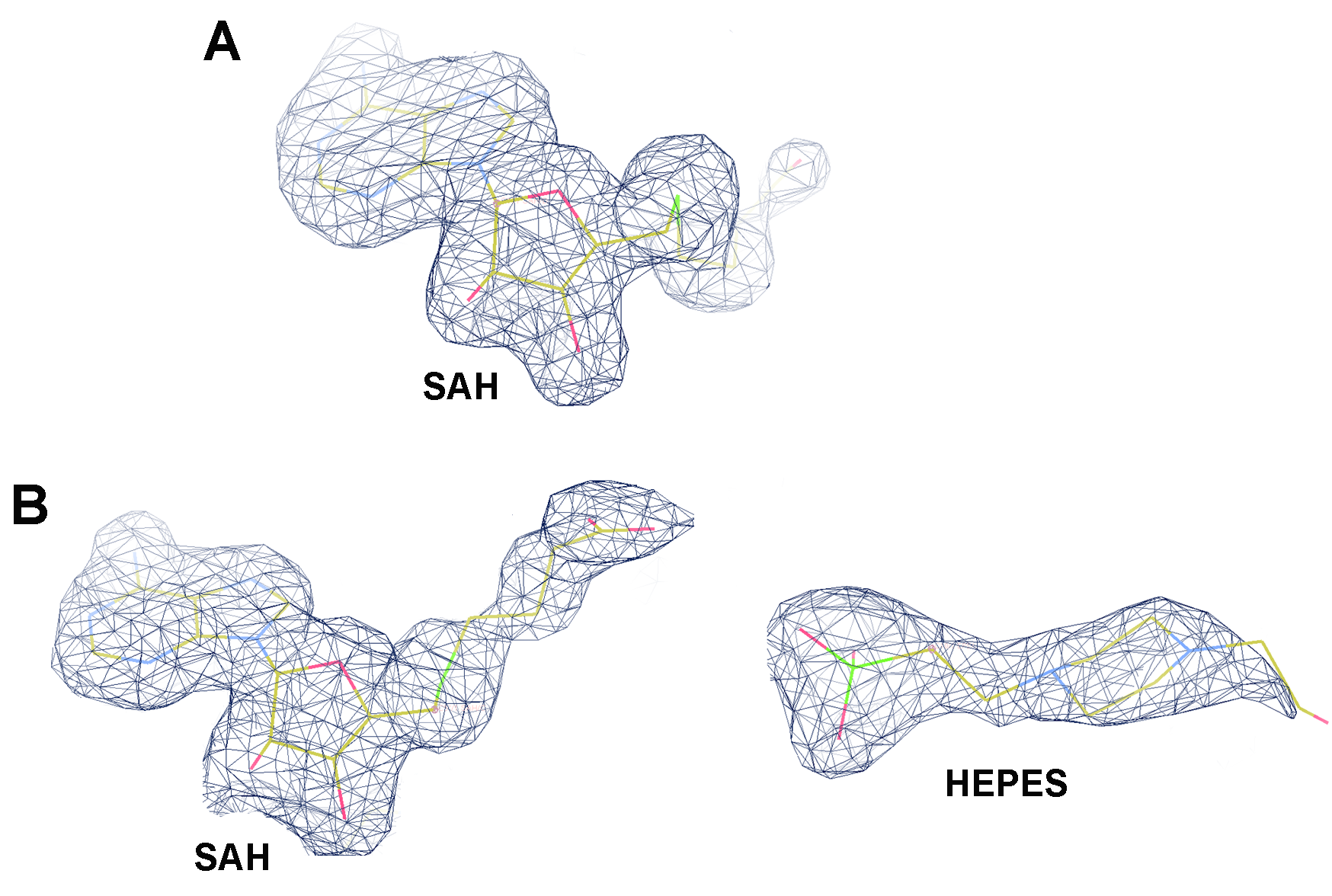


**Figure S2** The electron density map of ligands bound to *Ec*TrmL. (A) Electron density of SAH in subunit A of *Ec*TrmL. (B) SAH and HEPES molecules bound in subunit B. All the electron density maps are at contoured at 1.0 σ.

**Figure S3**

**
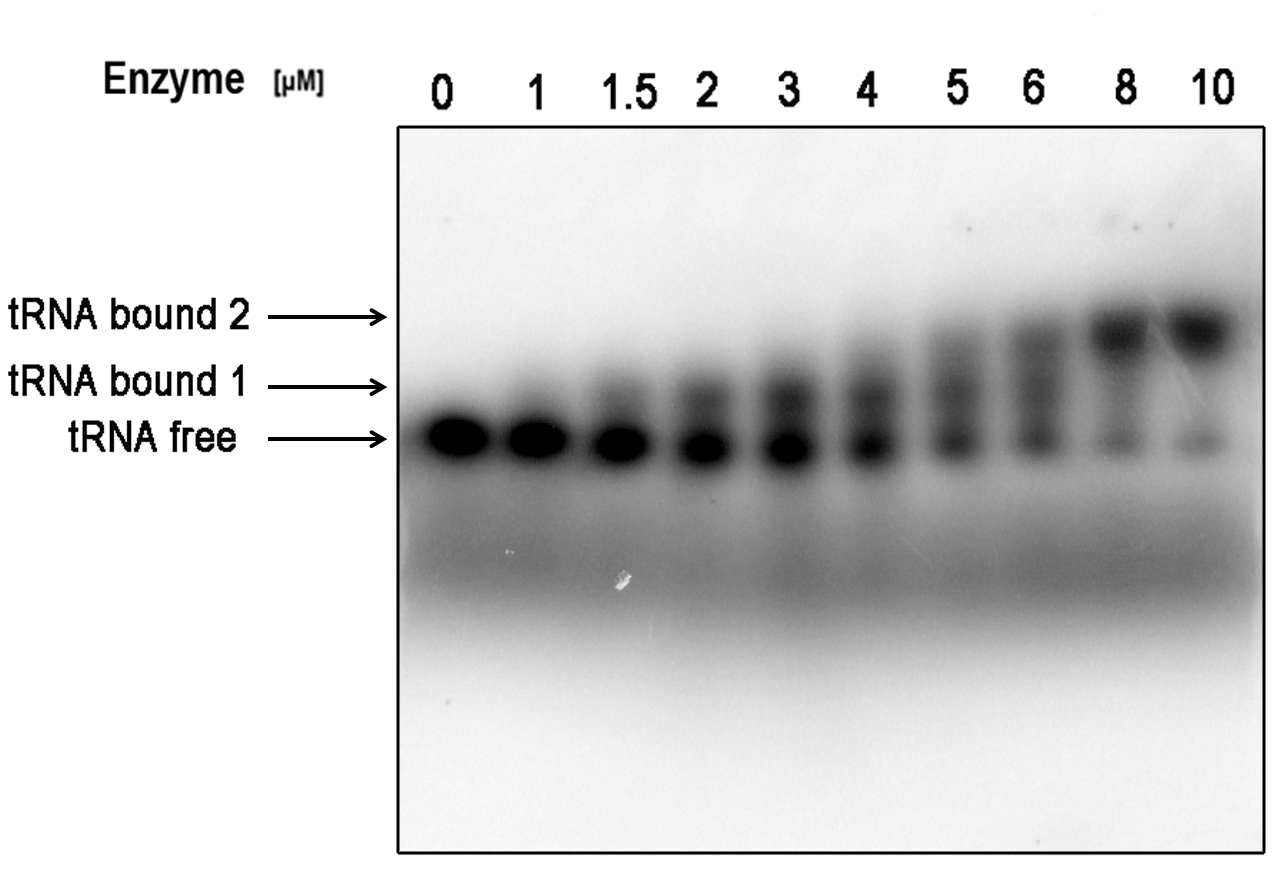
**

**Figure S3** The binding affinity of wild type *Ec*TrmL for tRNA analyzed by the gel mobility shift assay, 80nM 3'-^32^P-dTrmL_*Ec*tRNA^Leu^_CAA_ was used. The tRNA was labeled at the 3' end by the *E. coli* CCA-adding enzyme, using [α-^32^P]ATP.

**Figure S4**


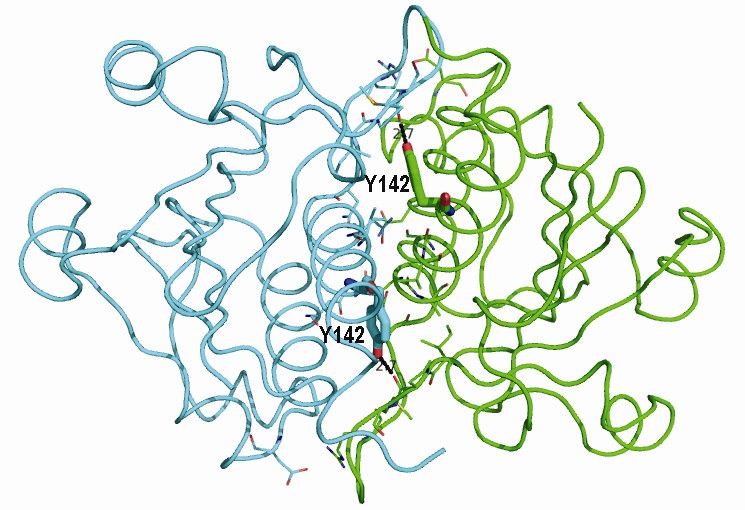


**Figure S4** The dimer interface of *Ec*TrmL. The structure of *Ec*TrmL dimer is shown in cartoon loop, the residues of the dimer interface are shown as sticks. Tyr142 is shown in thick sticks. Tyr142 is in the center of the dimer interface area and has multiple interactions with residues from the other subunit.

**Figure S5**


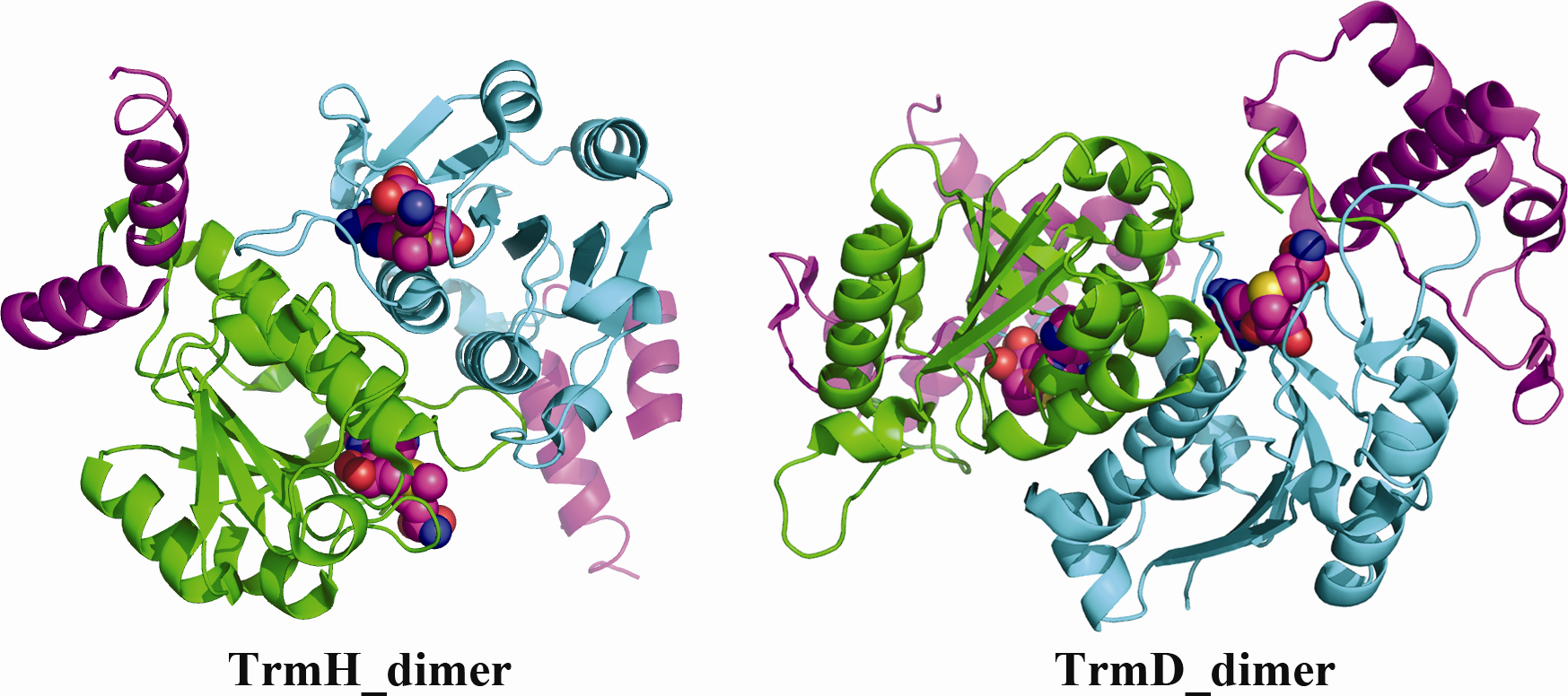


**Figure S5** Ribbon diagram showing the dimeric structure of TrmH and TrmD with one subunit colored in green and the other subunit in cyan. The extension domains are shown in magenta and the cofactors SAH or SAM are represented as spheres. The TrmH_dimer in complex with SAM is from *T. thermophilus* (PDB: 1V2X) and the TrmD_dimer bound with SAH is from *H. influenza* (PDB: 1UAL).
